# Inhibitors of Mammalian Aquaporin Water Channels

**DOI:** 10.3390/ijms20071589

**Published:** 2019-03-29

**Authors:** Mohammed Abir-Awan, Philip Kitchen, Mootaz M. Salman, Matthew T. Conner, Alex C. Conner, Roslyn M. Bill

**Affiliations:** 1 School of Life & Health Sciences, Aston University, Aston Triangle, Birmingham B4 7ET, UK; abirawam@aston.ac.uk (M.A.-A.); p.kitchen1@aston.ac.uk (P.K.); 2 Department of Cell Biology, Harvard Medical School, 200 Longwood Avenue, Boston, MA 02115, USA; Mootaz.Salman@childrens.harvard.edu; 3 Program in Cellular and Molecular Medicine, Boston Children’s Hospital, 200 Longwood Avenue, Boston, MA 02115, USA; 4 Research Institute of Health Sciences, School of Sciences, University of Wolverhampton, Wolverhampton WV1 1LY, UK; m.conner@wlv.ac.uk; 5 Institute of Clinical Sciences, College of Medical and Dental Sciences, University of Birmingham, Edgbaston, Birmingham B15 2TT, UK; a.c.conner@bham.ac.uk

**Keywords:** aquaporin, AQP inhibitors, AQP modulators, functional assays, AQP expression, AQPs in disease, AQP structure, TGN-020, heavy metals, small molecule inhibitors

## Abstract

Aquaporins (AQPs) are water channel proteins that are essential to life, being expressed in all kingdoms. In humans, there are 13 AQPs, at least one of which is found in every organ system. The structural biology of the AQP family is well-established and many functions for AQPs have been reported in health and disease. AQP expression is linked to numerous pathologies including tumor metastasis, fluid dysregulation, and traumatic injury. The targeted modulation of AQPs therefore presents an opportunity to develop novel treatments for diverse conditions. Various techniques such as video microscopy, light scattering and fluorescence quenching have been used to test putative AQP inhibitors in both AQP-expressing mammalian cells and heterologous expression systems. The inherent variability within these methods has caused discrepancy and many molecules that are inhibitory in one experimental system (such as tetraethylammonium, acetazolamide, and anti-epileptic drugs) have no activity in others. Some heavy metal ions (that would not be suitable for therapeutic use) and the compound, TGN-020, have been shown to inhibit some AQPs. Clinical trials for neuromyelitis optica treatments using anti-AQP4 IgG are in progress. However, these antibodies have no effect on water transport. More research to standardize high-throughput assays is required to identify AQP modulators for which there is an urgent and unmet clinical need.

## 1. Aquaporin Structure

**Figure 1:**
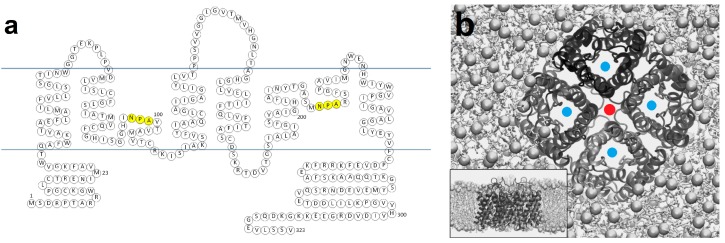
Aquaporin structure: (**a**) primary sequence of Aquaporin 4 (AQP4), the water channel with the highest recorded water permeability (see Table 1), highlighting the NPA (Asn-Pro-Ala) residues in yellow; (**b**) tertiary structure of the AQP4 homotetramer showing four water pores (marked with a blue dot) and the ‘fifth pore’ produced by formation of the tetramer (marked with a red dot). Adapted from an image originally published in [33].

Aquaporins (AQPs) are transmembrane proteins that facilitate the bidirectional transport of water across cell membranes; water flows through AQP pores following an osmotic gradient to support water homeostasis [1,2,3]. A subset of the AQP family, the aquaglyceroporins, also allows the transport of glycerol and other small neutral solutes such as urea and ammonia [4,5,6,7]. These include AQP3, AQP7, AQP9, and AQP10. Currently, there are 13 known mammalian AQPs, each with a structure comprising six membrane-spanning helices (Table 1). The amino- and carboxy-termini of APQs are both intracellular [3,8]. Two opposing, shorter helices do not span the entire membrane (Figure 1), but form the pore itself [3,9,10]. These half helices contain the family’s signature triplet amino acid NPA (Asn-Pro-Ala) motif, as seen in Figure 1a [8,11]. The residues in this motif orient water molecules to prevent protons from entering the channel, as well as acting as the hydrogen bond donors and acceptors that facilitate the passage of water molecules [3]. The narrowest segment of the AQP channel is within the transmembrane region of the pore. For AQP1, it is 2.8 Å in diameter, which is similar to the size of a single water molecule. This feature means water molecules pass through AQPs in single file. A high resolution (0.88Å) X-ray crystal structure of a yeast AQP, Aqy1, revealed that the selectivity filter within the pore contains four positions for water molecules and that it is not possible for these positions to be occupied simultaneously. Instead they travel in a pairwise fashion, filling two of these positions at a time [12].

AQPs form homotetramers within the plasma membrane. Each homotetramer consists of four water channels (Figure 1b) [13,14]. Each water channel is functionally independent. It has been shown that channel pore size can sometimes be variable. When the residues responsible for the widening and narrowing of the channel entrance were in an ‘open’ state, molecular dynamics simulations suggested that the channel entrance was wider in monomeric than tetrameric AQP5, although it is not clear how this might affect water permeability [15]. For AQP4, functional and mutagenesis studies suggested that non-tetrameric AQPs have the same water permeability as those found in tetramers [16]. Although the reason AQPs form tetramers is not fully understood [17,18], it has been shown that non-tetrameric forms of AQP4 do not undergo hypotonicity-induced intracellular trafficking [16]. 

A controversial topic in AQP biology, related to their homotetrameric nature, has been their conduction of ions and whether this takes place. AQP tetramers contain a potential fifth central pore as shown in Figure 1b, reminiscent of cation channels such as Kir 2.1 [19]. The use of forskolin has been reported to induce cationic permeability in AQP1 [20], however, follow-up experiments by different groups failed to replicate this [21]. Direct binding of cGMP to AQP1 expressed in *Xenopus* oocytes has been shown to cause an ionic current after cGMP binding to a predicted site found in the AQP1 carboxyl tail [22]. The central pore of AQP1 was also found to be gas permeable using molecular dynamics simulations. However, conduction of O_2_ and CO_2_ through AQP1 may only be physiologically relevant in membranes of low gas permeability where there are high concentrations of AQPs [23]. Recently, a review of evidence for the gas permeability of AQPs suggested that that it may be functionally important in some cells, but not in others [24]. Less controversially, AQP6 is permeable to nitrate and chloride ions [25,26,27,28], and AQP8 is permeable to ammonia [29,30,31]. In a high resolution X-ray crystal structure of human AQP5, however, a lipid molecule co-crystallized with the protein, occluding the fifth central pore, suggesting another possible role [32].

## 2. Aquaporin Function

AQP channels facilitate the movement of water and small uncharged solutes across membranes down a concentration gradient. As such, they are implicated in a myriad of diseases and AQP modulators are thought to be promising therapeutic targets for new drug development [8,14]. In some cases, compounds that modify the transcription and translation of AQPs may also be beneficial such as modifying AQP7 expression as a treatment for obesity [14]. Most of the information currently known about AQPs pertains to AQPs 1, 2, 3, and 4, which are the focus of this review. Table 1 shows the currently-known structural and functional features of AQP family members. AQP4 appears to have the highest known water permeability of the AQPs and its role as the major AQP within the central nervous system (CNS) supports this, as tight control is required to ensure optimal conditions for CNS function. The lowest permeabilities are seen in AQP0 and AQP6. The main function of AQP0 as the major intrinsic protein (MIP) in the eye lens (where it has a structural and adhesive function) means that it does not require a high water permeability. AQP6 is an intracellular AQP, but its main function is not yet known.

### 2.1. AQP1

AQP1 was first identified in red blood cells and is linked to pathologies such as cancer, the inability to concentrate urine, and altered pain sensation [37,40,56]. Expression of AQP1 in some tumor types is correlated with increased angiogenesis, invasion, metastasis, and growth [3,57]. Aggressive melanomas were shown to have higher expression of AQP1. Silencing of the AQP1 gene in a mouse melanoma xenograft model, reduced angiogenesis and metastasis [1]. In addition to this, it was found that matrix metalloproteinase (MMP) 2 was downregulated upon AQP1 knockdown although it was not determined which of AQP1 or MMP2 was important in mediating this effect [1]. AQP1 is also found in the endothelial cells of the lungs and epithelial cells of bile ducts as well as the kidney tubules and in the epithelia of the proximal convoluted tubule and the loop of Henle [10,47]. AQP1-deficient humans are unable to concentrate their urine beyond 450 mOsm compared to the more normal 1000 mOsm and above [8,58]. They are also prone to fluid accumulation in the lungs [47]. Modulating AQP1 may therefore lead to treatments that can aid conditions of excess fluid such as heart failure and cirrhosis [3,10,14]. Another role of AQP1 is pain perception; its deletion can cause a reduction of pain from inflammation or cold providing a possible basis for analgesia [3]. There is also evidence to show AQP1 in the choroid plexus where cerebrospinal fluid is produced [47]. 

### 2.2. AQP2

AQP2 is found in the kidneys, specifically within the cells of the collecting duct. Its regulation is one of the best-studied examples of hormone-mediated AQP translocation; following vasopressin (anti-diuretic hormone) release, AQP2-containing vesicles translocate to the plasma membrane facilitating water reabsorption [8,59]. Vasopressin binds to V_2_ receptors of the principal cells in the renal collecting ducts, activating cAMP and protein kinase (PK) A, which phosphorylates Ser-256 and causes the exocytosis of AQP2-containing vesicles [25,60]. Central diabetes insipidus is the lack of vasopressin and its key symptoms are polyuria with an inability to concentrate urine [61]. Nephrogenic diabetes insipidus (NDI) is usually caused by a problem with the vasopressin receptor on collecting duct cells and is often linked to the AQP2 gene expression [61]. There are also cases of inherited NDI which can be caused by the same vasopressin receptor mutations but also mutations in the AQP2 gene; 19 such mutations have been identified [42,60]. Acquired diabetes insipidus is seen in cases such as long term lithium therapy where AQP2 subcellular localization can become dysregulated resulting in reduced surface localization and therefore loss of AQP2 function [47]. When vasopressin is secreted inappropriately, high levels of water retention and hyponatremia occur [61]. This is also seen in conditions such as congestive heart failure, where there is increased water retention due to an upregulation of vasopressin, and consequently AQP2 expression and translocation to the plasma membrane [60,62]. The exact mechanism by which AQP2 is recycled in the principal collecting duct cells is not yet known, however clathrin-coated pits are thought to have a role in this process [63]. 

### 2.3. AQP3

AQP3 is an aquaglyceroporin that allows the transport of glycerol in addition to water [8]. It is found in the epithelia of renal collecting ducts, urinary tract, digestive tract, and respiratory tract [47]. It is also found in the skin and aids skin hydration [13]. The overexpression of AQP3 is linked to skin malignancies such as melanomas [64,65]. AQP3 knock-out (KO) mice have been shown to be resistant to tumor formation in skin cancer models through reduced glycerol transport, resulting in lower ATP formation and reduced cell proliferation [3,14,57,64]. AQP3 has also been shown to transport hydrogen peroxide and this function aids NF-κB signaling. AQP3 KO mice had reduced H_2_O_2_ levels, impaired NF-κB activation and a reduced induction of psoriasis [66]. In addition to this, AQP3 has roles within the normal function of bacterial phagocytosis by macrophages and the trafficking of T cells [3,67,68].

### 2.4. AQP4

AQP4 is the main water channel expressed in the CNS and is found at highest concentration in astrocyte end-feet, which together with blood vessels form the blood–brain barrier (BBB) [13,69]. AQP4 expression is increased in cerebral edema and ischemia. This has been shown to be correlated with the release of inflammatory cytokines [3,13,70,71]. AQP4 is also thought to aid the migration of astrocytes when neuronal injury occurs and glial scars form [72]. AQP4 has been shown to have a link with seizures where the regulation of cell volume by water flow can reduce susceptibility to seizures [73]. For the potassium channel, Kir 4.1, to be active, water flow through AQP4 is required [1,9,71,74,75]. During trauma or ischemia to the brain, there is a state of hypoxia which is then followed by a failure in Na^+^, K^+^, and Cl^−^ pumps in the plasma membrane, causing dysregulation of osmolality [56,76,77,78]. This results in an increased intracranial pressure (ICP) and cerebral edema due to water flow, primarily into astrocytes during the cytotoxic phase of cerebral edema [70]. Limited therapeutic options are currently available to control cerebral edema. They are restricted to the administration of hyperosmotic solutions (such as mannitol, glycerol, and sodium chloride), hypothermia, or ultimately a surgical intervention to remove part of the skull to alleviate the uncontrolled rise in ICP [70,79,80]. AQP4 inhibition may be beneficial in cytotoxic edema as AQP4 KO mice were protected from cytotoxic edema. [3,13,14,56,70,81]. Vasogenic edema occurs due the movement of water and plasma proteins across a leaky BBB [9,76]. In models of vasogenic edema, however, AQP4 KO resulted in worse outcomes, due to an inability to remove excess water from brain tissue [3,9,56,70]. Any inhibition of AQP4 function, therefore needs to be acute. 

Neuromyelitis optica (NMO) is an autoimmune condition where the CNS is affected by inflammation and demyelination [82]. It is symptomatically similar to multiple sclerosis (MS), but there are some key differences including the fact that some treatments for MS can exacerbate NMO [82]. Most patients with NMO have autoantibodies that target AQP4 in astrocytes and cause complement- and cell-mediated cytotoxicity [83]. The usual treatment for NMO involves steroids and other immunosuppressants such as azathioprine and mycophenolate [82]. 

### 2.5. AQPs 0, 5–12

AQP0, also known as MIP, was first identified within the lens. Its role is not only as a water channel (it has a low permeability compared to other family members, Table 1) but also to maintain the transparency and structure of the lens [35]. Mutations in the genes for AQP0 were found to result in congenital cataracts [35,84]. 

AQP5 was first found within the submandibular glands and is now known to have a role within many secretory glands including salivary, lacrimal, and sweat glands [85]. Binding of acetylcholine to muscarinic M3 receptors is thought to induce the translocation of AQP5 vesicles to the cell surface membrane and problems with this process have been shown to be linked to conditions such as Sjögren’s syndrome [32,86,87].

AQP6 is an intracellular water channel found in vesicles and its water permeability is low [48], as shown in Table 1. These vesicles also contain H^+^–ATPase and AQP6 has been found to become more active at low pH suggesting that the role of AQP6 is in acid secretion [27,84].

AQP7, an aquaglyceroporin, is found in adipocytes and is thought to have a role in fat metabolism aiding degradation of triglycerides in fasting states [8]. AQP7 KO mice are seen to have higher levels of fat than wild type controls and this is thought to be a therapeutic target for obesity [13]. However, in the case of an individual with a homozygous nonfunctional AQP7 mutation, body mass index, cholesterol, and glucose levels were all normal, with only a reduced increase in glycerol levels during exercise compared to controls [88].

AQP8 has been identified in the brain, small intestine, testis, and the salivary glands [49]. Its function was disputed, but it is now known that AQP8 transports ammonia, as well as water, and that the transport of ammonia may be its primary function, maintaining acid-base equilibrium [30].

AQP9, an aquaglyceroporin, is found in hepatocytes and its function is thought to be significant during periods of exercise or prolonged fasting affecting glucose production [85]. It is found in osteoclasts and is thought to have a role in osteoporosis [89]. It is also found in astrocytes and leukocytes [43,49]. AQP9 and AQP7 are also known to mediate the uptake of arsenic compounds, which are used in some cancer treatments [44].

Little is known about the remaining AQPs. AQP10, an aquaglyceroporin, is found in intestinal epithelial cells and in adipocytes [43,47,90]. AQP11 is an intracellular aquaporin but its function remains unclear [44]. AQP11 contains a cysteine residue instead of an alanine in its first NPA motif and in the second motif, it has a leucine instead of an arginine [91]. It is found to be expressed in the testis, brain, kidneys, and liver [43]. AQP11 KO mice have been found to die soon after birth due to polycystic kidney disease [92]. This is thought to be due to AQP11 being required for correct glycosylation of polycystin-1 and -2 [93]. AQP12 is expressed in pancreatic acinar cells and in AQP12 KO models, those that develop pancreatitis develop a much more severe pathology [54,55].

## 3. Aquaporin Assays

Water permeability can be assayed in various ways. Measuring volume change due to osmotic gradients is one indirect approach. Epithelial assays are based on using epithelial tissues placed on permeable supports and adding solutes to cause the fluid in a capillary tube to move. Transepithelial flux is measured via this change and fluorescent dyes can be used to evaluate permeability [94,95]. Transepithelial permeability results from water flow that has occurred both cellularly and paracellularly, whereas water flux through AQPs occurs only cellularly, which is a limitation of this as an approach to deducing AQP function. 

Osmotic swelling techniques are by far the most common methods used for assaying volume changes and *Xenopus* oocytes are often used as they have a very low intrinsic water permeability [96]. Osmotic gradients are used and oocytes expressing AQPs change volume when these gradients are altered [97]. While the use of *Xenopus* oocytes is very common, there is substantial variability between oocyte preparations, meaning data may vary between laboratories even using the same materials [94,98]. 

Microscopy techniques are also used to measure permeability via a fluorophore that is taken up by cells. Esterases within the cell produce the active fluorophore and the rate of change of fluorophore concentration can then be detected using a plate-reader or fluorescence microscope after an osmotic gradient has been applied [99]. The limitation of this technique is that the fluorescence is relative to the dimensions of the cell monolayer where thinner cells produce a larger relative change in fluorescence than cell-lines that have a thicker monolayer. This can affect reproducibility in the same cell-line if confluence is varied between assays. It is also dependent on the amount of fluorophore that is taken up by the cells, which can sometimes be pumped out, producing variability in the data [100]. 

Stopped-flow spectroscopy involves the use of suspensions of cells, vesicles, or proteoliposomes which are mixed with solutes to produce a change in volume. The difference in light-scattering after this volume change can then be used to calculate the volume change [99]. Limitations of stopped-flow spectroscopy techniques include the dead time and mixing ability of the instrument used [101,102]. 

Computational methods involve the use of high-resolution structures and molecular dynamics simulations, which can provide new insights into AQP structure and function at atomic resolution. However, high-quality structures are not always available and simulations can sometimes produce data that are different from experimental data [94,103]. A summary of these techniques is shown in Table 2. 

## 4. Aquaporin Inhibitors

Most of the molecules that are currently under investigation as AQP inhibitors target AQPs 1, 2, 3, or 4. There are many patents, clinical trials, and studies on AQP up-regulators, modulators, and inhibitors. For the purposes of this review, only AQP inhibitors are considered (Table 3).

Several molecules have been suggested as inhibitors of various AQPs over the past few years. Of these, most are small molecules (Table 3). The similarity found between AQPs and ion channels has caused interest in ion channel modulators as possible AQP inhibitors [43]. Arylsulfonamides such as acetazolamide are molecules that have attracted attention as potential AQP inhibitors [122]. Anti-epileptic drugs (AEDs) have also been suggested to have an AQP modulating function [70]. However, they have yet to be proven as definitive AQP inhibitors. A novel molecule named TGN-020 has shown great promise very recently as an inhibitor in mouse models [81]. Micro RNA (miRNA) and small interfering RNA (siRNA) have also been used to modulate AQPs [70]. Mercury chloride binds to a pore-lining cysteine residue found on several (but not all) AQPs and blocks the channel [3]. Mercury and its compounds is known to be toxic due to their non-specificity which causes many off-target effects [11]. Other heavy metal compounds (such as silver and gold compounds) have been investigated as inhibitors, but the challenge is finding a molecule with a side-effect profile that is tolerable. Currently-known inhibitors are discussed by type in the sections below.

### 4.1. Small Molecule Inhibitors

Tetraethylammonium (TEA) is a small molecule inhibitor that was first tested due to perceived similarities between AQPs and ion channels. Using human AQP1 expressed in *Xenopus* oocytes, a maximum single dose of 10 mM TEA caused a reduction in water permeability of 33% [105]. Another study using 4 µM and 100 µM TEA showed an inhibition of 44% for *Xenopus* oocytes expressing AQP1, with no differences at the two concentrations; TEA also inhibited AQP2 and AQP4, with maximal inhibition of 40% and 57%, respectively, at 100 µM TEA [123]. Further testing of TEA in *Xenopus* oocytes or in native AQP1-expressing erythrocytes has failed to show inhibition [3,124]. TEA is thought to act by binding to a tyrosine residue located on the extracellular end of transmembrane helix 5 [123].

Acetazolamide is a carbonic anhydrase inhibitor used in glaucoma to reduce aqueous humor production and hence intraocular pressure [125]. It has been shown to be a reversible inhibitor of AQP1 and AQP4 (Table 3). It has inhibitory effects on *Xenopus* oocytes expressing human AQP4 in a dose-dependent manner with maximal inhibition of 85% at the highest dose of 20 µM [126]. A reduction in the water permeability of rat AQP4 (assayed in proteoliposomes) to 46% was observed at a maximum dose of 1.25 mM acetazolamide [127]. In HEK293 cells expressing rat AQP1, there was a 39% reduction in water permeability with 100 µM acetazolamide compared to untreated rat AQP1 (using GFP fluorescence to measure swelling) [119]. However, acetazolamide appears to be unable to inhibit endogenous AQPs in human cells [10,124].

Anti-epileptic drugs (AEDs) have also been suggested as possible AQP inhibitors [120]. Their anti-epileptic action has been hypothesized take place through modulation of AQPs. Many AEDs such as topiramate, zonisamide, and lamotrigine are known to have a similar inhibitory effect to acetazolamide on carbonic anhydrase enzyme as well as an in silico predicted binding site on AQP4 that is similar to that hypothesized for acetazolamide [120]. AEDs had an inhibitory effect on AQP4 in *Xenopus* oocytes [120], however, this could not be reproduced in rat thyroid epithelial cells [122]. AEDs are thought to possibly have an inhibitory effect on AQP1, AQP4, and AQP5 [128]. However due to their unconfirmed mechanisms of action and relatively non-specific action, there is no conclusive evidence showing that AEDs are effective and safe AQP inhibitors [10,122].

TGN-020 was shown in mouse models to significantly reduce AQP4-mediated edema following ischemia, but the molecule was administered before injury [81]. There was an approximately 10% reduction in brain volume [81]. Therapeutic administration must necessarily occur after a stroke, although a prophylactic treatment could be considered if the side effect profile of TGN-020 was acceptable. TGN-020 has since been tested on the same middle cerebral artery occlusion (MCAO) model and administered post-injury in mice. In a study using mouse models at a dose of 100 mg/kg, TGN-020 was administered 15 minutes post-ischemic injury and treated animals had better motor scores and less AQP4 expression around blood vessels when compared to untreated controls [129]. The half-maximal inhibitory concentration (IC_50_) of TGN-020 was 3µM in *Xenopus* oocytes expressing human AQP4, but there is no evidence to show that TGN-020 is AQP4-specific [130].

Phloretin is a small molecule that acts as a non-specific aquaglyceroporin inhibitor. It has also been shown to inhibit the urea transporter, UT-A1, found in the kidney [131]. It has been speculated that the same mechanism underpinning urea inhibition is responsible for inhibition of the aquaglyceroporins, AQP3 and AQP9 [107,132]. Then 100 µM of phloretin was used to inhibit AQP9 expressed in *Xenopus* oocytes, resulting in an 86% inhibition [107]. AQP3 glycerol permeability in proteoliposomes was inhibited by 500 µM phloretin, producing 83% inhibition. Phloretin had no effect on control proteoliposomes which had a P_gly_ of ~2.8 × 10^−6^ cm/s [108].

A recent study identified new inhibitors of the aquaglyceroporins, AQP3 and AQP7. The compound DFP00173 was able to inhibit the glycerol permeability of human erythrocytes with an IC_50_ of ~0.2 µM. Compound Z433927330 reduced glycerol permeability with an IC_50_ of ~0.6 µM. In a Chinese hamster ovary cell-line, compound DFP00173 was able to inhibit mouse AQP3 with an IC_50_ of ~0.1 µM and was selective for AQP3 over AQP7 and AQP9. Compound Z433927330 was able to inhibit mouse AQP7 in the same cell-line with an IC_50_ of ~0.2 µM and with selectivity for AQP7 over AQP3 and AQP9; IC_50_s for this compound for mouse AQP3 and AQP9 were ~0.7 µM and ~1.1 µM, respectively [121].

### 4.2. Heavy Metal Ion Inhibitors

Heavy metal compounds have been used in cytotoxic treatments for many years, with the use of the platinum compound cisplatin being notable in cancer therapy. Many AQPs have been correlated with cancers, their presence influencing disease severity, increased local invasion, and the occurrence of metastasis [57]. AQP1 overexpression has been identified in brain, breast, lung, renal, cervical, ovarian, and colorectal cancers [133]. AQP3 overexpression has been found in skin, stomach, renal, liver, colorectal, lung, and cervical cancers [134]. AQP3 is highly expressed in skin carcinoma cells. The uptake of glycerol through AQP3 is thought to aid the growth of these cancer cells [57]. AQP4 overexpression is found in brain, lung, and thyroid cancers [135]. AQP5 overexpression has been found in breast, cervical, colorectal, liver, lung, pancreatic, ovarian, and esophageal cancers [136]. AQP7 and AQP8 overexpression are found in thyroid and cervical cancer, respectively [133]. AQP9 overexpression has been found in brain, liver, and ovarian cancers [136].

Nickel chloride has been shown to inhibit AQP3 overexpressed in human bronchial epithelium cells. Water permeability was reduced to approximately 60% in cells treated with 1 mM NiCl_2_ compared to non-transfected control cells [114]. Mutational studies showed that the extracellular loop residues, Trp-128, Ser-152, and His-241, were all required for the observed inhibitory effect, but that further studies are required to confirm similar effects in other AQP3-expressing tissues such as the kidney [114]. Copper ions also inhibit AQP3 function and attempts to reduce the toxicity of copper compounds have been addressed by using nanoparticles for delivery [57]. Copper sulfate has been identified as an AQP3 inhibitor at 100 µM [113]. It is believed that copper ions act by binding the same three residues on the extracellular loops as nickel ions [137]. This inhibition mechanism is different from that of mercury ions, which bind to Cys-40 in AQP3 [137,138]. Copper compounds have been shown to specifically inhibit AQP3 and not AQP4, AQP5, or AQP1 [137], while other AQPs remain to be tested. Gold-based compounds have also been used as AQP3 inhibitors and have been shown to be effective. AQP3 is found in erythrocytes, alongside AQP1, however its role is mostly for glycerol rather than both water and glycerol permeability [110]. Erythrocytes showed a 90% decrease in glycerol permeability following treatment with 50 µM AuPhen. The binding of this gold compound is thought to be via Cys-40 and inhibition can be almost completely reversed by the addition of the reducing agent, 2-mercaptoethanol [110]. AuPhen and other Au(III) compounds such as Auterpy were also compared with copper and platinum compounds as AQP3 inhibitors [111,139]. Due to their effective inhibition of glycerol transport in AQP3, they were tested on AQP7, another aquaglyceroporin. Initial results have demonstrated that 15 µM AuPhen inhibited water and glycerol transport through AQP7 [111]: there was a reduction in water permeability (63%) as well as glycerol permeability (79%). Adipocytes overexpressing hAQP7 were used and permeability was measured using loading with 5 µM of calcein-AM and hyperosmotic shock. In silico docking studies suggest that Auphen binds AQP7 through an interaction with Met-47 [140]. Silver has also been used as a possible AQP inhibitor and is found to produce a rapid and irreversible inhibition of AQP1 in erythrocytes [112]. It has been shown to be a much more potent inhibitor of AQP1 in erythrocytes than mercury, [112]. Silver nitrate and silver sulfadiazine were tested as inhibitors to prevent shrinking of human erythrocytes in hyperosmotic solutions. A dose-response curve was used to calculate an IC_50_ of 3.9 µm and 1.24 µm and a 60% and 75% inhibition for silver nitrate and silver sulfadiazine, respectively. Gold and silver are thought to bind to sulfhydryl groups on cysteine residues of AQPs, but the complete mechanisms are not yet understood [110,111,112].

## 5. Antibody Treatments

An anti-AQP4 monoclonal antibody was developed as a potential treatment for NMO. “Aquaporumab” competitively binds to AQP4 in astrocytes and displaces AQP4-IgG, reducing NMO lesions in mouse models [82,83]. This is yet to be tested in humans, although in vivo studies have been promising [141].

## 6. Patents and Clinical Trials

A search of the European Patent Office archive in February 2019 returned 484 AQP-related patents or patent applications. These documents protect various techniques and methods such as AQP upregulation and modulation, detection methods, mRNA expression, as well as siRNA silencing techniques. Approximately 40 of these patents are for inhibitors of AQPs or NMO treatments.

US patent 2008/0194513 A1 identifies the proliferation of blood vessels within the eyes as a factor in sight loss in several ocular conditions. Interfering RNAs were therefore used to silence AQP4 mRNA expression in order to reduce ocular neovascularization. Administration of siRNA at 1–10 nM provided over 70% inhibition of AQP4 expression in Madin-Darby canine kidney (MDCK) cells transfected with human AQP4 compared to controls [142]. A similar patent was filed by the same inventors using similar techniques for the inhibition of AQP1 expression for ocular conditions [143].

US patent 7,659,312 B2 identifies various AQP4 inhibitors to be used in cerebral edema and other water-related disorders. Twenty-one compounds are described that are similar in structure to the loop diuretics, furosemide, and bumetanide. A total of 15 compounds had inhibitory effects on AQP4 when tested in *Xenopus* oocytes transfected with AQP4-M23. 20 µM inhibitor compounds were used and experiments were completed twice. Aryl sulphonamide-like compounds and bi-aryl compounds were identified as inhibitors. Docking energies were also analyzed using AQP4 monomers built from electron diffraction structures; compounds with higher inhibition often had better docking energies.

Phenylbenzamide-based compounds are patented inhibitors of AQP2 and AQP4 for the treatment of AQP-related conditions such as edema, ischemic stroke, epilepsy, and meningitis. Phenylbenzamides such as niclosamide, a compound usually used for treatment of helminth infections, is known to have an inhibitory effect on NF-kB and IKK-β. The compound IMD-0354 was used to inhibit IKK-β in myocardial ischemia models [144]. Compound 3 has a similar structure to IMD-0354 but the two trifluromethyl groups are replaced by chloride. Compound 3 is claimed to be an inhibitor of AQP2, AQP4-M1, and AQP4-M23 with over 60% inhibition. It was at very poor inhibitor of AQP1 and AQP5. Compound 1 (IMD-0354) inhibited cerebral edema formation in a murine water toxicity model. Treatment with 0.76 mg/kg produced an 11.2% reduction, while 7.6 mg/kg showed a 15.9% reduction in edema. Following MCAO in mice, there was a 29.4% increase in survival 24 h post-injury [118]. A subsequent patent described prodrug salts of these compounds [145].

Ghrelin is a 28 amino acid peptide that is secreted by gastric mucosa cells and is a hormone that regulates hunger. It is known to also have other anti-inflammatory properties thought to be protective in neuronal injury [146]. Traumatic brain injury (TBI) causes acute, elevated ICP and brain damage. Repeated mild brain injuries (mBI), such as concussions, can cause long-term injury to the brain and increased risk of neurodegeneration with aging. Treatments for TBI and mBI are very limited. Administration of ghrelin reduced the levels of reactive oxygen species in neurons and volume loss in mice post-injury [147]. The administration of ghrelin at doses 1.5–1000 times native levels provided therapeutic effects lasting from 30 minutes up to 24 h. Ghrelin was also able to reduce oxidative bursts post mBI [148]. Ghrelin has been shown to reduce levels of brain water after acute hypoxia in rats, as well as the permeability of blood vessels. It is thought to do this through reduction in TNF-α levels together with other anti-inflammatory effects on IL1, IL6, and NF-κB [149]. In another study, levels of AQP4, serum S100B and vascular permeability were quantified to show disruption of the BBB post-TBI in mice. Treatment with ghrelin was able to reduce these levels and help prevent BBB damage post-TBI [150].

A possible NMO treatment centers on a peptide corresponding to AQP4 loop C. T cells reactive to this peptide from AQP4 -/- mice produced a pathological response reminiscent of NMO when injected into AQP4 +/+ mice. T cells are found in NMO lesions and are thought to be required for the production of anti-AQP4 IgG. It was noted that immunosuppression therapy would be required before treatment takes place [151].

US patent 2015/0224108 A1 presents techniques to create prodrugs and derivatives of the loop diuretics, bumetanide and furosemide, as AQP inhibitors and to aid their administration to the site of action [152].

The use of phloretin as an AQP9 inhibitor was shown to reduce osteoclast levels. This inhibition would aid treatment in conditions such as osteoporosis, where excessive bone resorption takes place. AQP9 expression is higher in osteoclasts than other cells; 50 µM phloretin inhibited AQP9 expression, causing a reduction in size and number of osteoclasts but not their precursors [153].

A search of the US National Library of Medicines Clinical Trials archive in February 2019 returned 61 clinical trials involving AQP-based treatments. Several of these trials examine urinary AQP2 levels and the conditions under which they are altered. Several trials also examine NMO, MS, and the therapeutic use of AQP4-IgGs. A search of the University Hospital Medical Information Network (UMIN) - Clinical Trials Registry in February 2019 returned two current clinical trials taking place using anti-AQP4 IgGs. There are currently no clinical trials taking place in the UK using AQP inhibitors or anti-AQP4 IgGs according to the UK Clinical Trials Gateway.

## 7. Discussion

Finding inhibitors for AQP water channels has been considerably more challenging than first thought. The identification of molecules that inhibit water transport through specific AQP isoforms is not a simple proposition for drug discovery on account of the high level of structural conservation within the AQP family (Table 1). The small diameter of all AQP-pores together with the chemical properties of the pore-lining amino acid side-chains mean that hydrophilic compounds are unlikely to enter and block them.

No definitive, small molecule AQP inhibitors have been identified to date that could be used therapeutically. Heavy metal compounds are certainly effective AQP inhibitors, but their non-specificity and toxicity substantially limit their therapeutic potential. Using liposomes to administer heavy metal compounds would reduce off-target effects, but more research is required. The most promising AQP inhibitor with clinical potential to date is TGN-020, which has not yet shown any side effects in vivo, but is yet to be tested in human clinical trials. Notably, antibodies used for NMO treatment have no effect on water permeability. There is a clear need for AQP modulators, particularly in conditions such as TBI where, in the early stages, AQP4 inhibition is required to manage cytotoxic edema but, thereafter, upregulation of AQP4 would aid clearance of vasogenic edema.

The lack of reliable in vitro phenotypic assays suitable for screening and validating the pharmacological regulation of AQP function (Table 2), means that pharmaceutical companies have been unable to meet the challenge of developing small molecules that block the AQP pore. For example, molecules discovered using *Xenopus* oocytes consistently fail to show efficacy in mammalian cells and vice versa. Screening programs for AQP inhibitors should therefore include multiple assays that allow more robust and reproducible readouts.

## 8. Conclusions

AQPs are expressed ubiquitously and have implications in myriad human diseases. The progress of identifying AQP inhibitors has been slow and as research continues, AQPs are increasingly implicated in more diseases, highlighting the clear and urgent need for AQP modulators. More research is needed to find effective AQP inhibitors. Current screening processes are extremely variable. Approaches to standardize assays would be beneficial in identifying promising molecules for future study.

## Figures and Tables

**Table 1:** A summary of AQPs 0–12 showing the information available on their structure, permeability, location within the body and pathophysiology. The osmotic water permeability coefficient (P_f_) relates the magnitude of bulk water flow across a membrane in response to hydrostatic or osmotic pressure gradients to the magnitude of the driving pressure gradient. It depends on both the permeability of individual water channels and the density of channels in the membrane. P_gly_ is the glycerol permeability coefficient. The single channel osmotic water permeability coefficient (p_f_) is also given where available.

AQP	Structural Information Available	Water Permeability (Glycerol Permeability Is Also Given for Aquaglyceroporins)	Main Locations in Humans	Disease States	References
AQP0	PDB ref: 1YMG Resolution: 2.2 Å Species: *Bos taurus* Method: X-ray diffraction Amino acids: 263	*Xenopus* oocytes—5 ng cRNA P_f_: 1.3 × 10^−3^ cm/s (control with no AQP 0.67 × 10^−3^ cm/s) p_f_: 0.25 × 10^−14^ cm^3^/s—single channel permeability	Lens	Mutations in the gene for AQP0 often result in bilateral cataracts	[7,34,35,36]
AQP1	PDB ref: 1FQY Resolution: 3.8 Å Species: *Homo sapiens* Method: Electron crystallography Amino acids: 269	*Xenopus* oocytes—5 ng cRNA P_f_: 19 × 10^−3^ cm/s (control 0.67 × 10^−3^ cm/s) p_f_: 6.0 × 10^−14^ cm^3^/s—single channel permeability	Erythrocytes Choroid plexus Eye epithelium Corneal epithelium Bile duct Capillaries Nephron Airways Skeletal muscle	Deficiency causes inability to concentrate urine Tumors with AQP1 have increased metastases and invasiveness Altered pain sensation	[3,7,37,38,39,40]
AQP2	PDB ref: 4NEF Resolution: 2.75 Å Species: *Homo sapiens* Method: X-ray diffraction Amino acids: 242	*Xenopus* oocytes—5 ng cRNA P_f_: 10 × 10^−3^ cm/s (control 0.67 × 10^−3^ cm/s) p_f_: 3.3 × 10^−14^ cm^3^/s—single channel permeability	Collecting duct cells	Deficiency causes diabetes insipidus	[7,39,41,42]
AQP3	No structural data available UniProtKB—O92482 Species: *Homo sapiens* Amino acids: 292	*Xenopus* oocytes—5 ng cRNA P_f_: 8.0 × 10^−3^ cm/s (control 0.67 × 10^c3^ cm/s) p_f_: 2.1 × 10^−14^ cm^3^/s—single channel permeability P_gly_: 23 × 10^−6^ cm/s	Epidermis Collecting duct cells Erythrocytes	Skin dehydration Psoriasis Tumor invasiveness and metastases	[7,43,44,45]
AQP4	PDB ref: 3GD8 Resolution: 1.8 Å Species: *Homo sapiens* Method: X-ray diffraction Amino acids: 223	*Xenopus* oocytes—5 ng cRNA P_f_: 29 × 10^−3^ cm/s (control 0.67 × 10^−3^ cm/s) p_f_: 24 × 10^−14^ cm^3^/s—single channel permeability	Astrocytes Parietal cells Collecting duct cells Retina	Cytotoxic edema Vasogenic edema Neuromyelitis optica	[7,44,46]
AQP5	PDB ref: 3D9S Resolution: 2 Å Species: *Homo sapiens* Method: X-ray diffraction Amino acids: 266	*Xenopus* oocytes—5 ng cRNA P_f_: 10 × 10^−3^ cm/s (control 0.67 × 10^−3^ cm/s) p_f_: 5.0 × 10^−14^ cm^3^/s—single channel permeability	Salivary, lacrimal and sweat glands Lung	Sjögren’s syndrome	[7,32,47]
AQP6	No structural data available UniProtKB—Q13520 Species: *Homo sapiens* Amino acids: 282	*Xenopus* oocytes—5–10 ng cRNA P_f_: 1.2 × 10^−3^ cm/s (control 0.53 × 10^−3^ cm/s) (93 × 10 ^−4^ cm/s after activation with mercury) No single channel data available.	Collecting duct	Unknown	[48,49]
AQP7	No structural data available UniProtKB—O14520 Species: *Homo sapiens* Amino acids: 342	*Xenopus* oocytes—5 ng cRNA P_f_: 18.6 × 10^−3^ cm/s (control 1.7 × 10^−3^ cm/s) P_gly_: 18.9 × 10^−6^ cm/s No single channel data available	Adipocytes Testis Proximal kidney tubule	Adipocyte hypertrophy	[4,8]
AQP8	No structural data available UniProtKB—O94778 Species: *Homo sapiens* Amino acids: 261	*Xenopus* oocytes—10 ng cRNA P_f_: 22 × 10^−3^ cm/s (control 0.8 × 10^−3^ cm/s) p_f_: 8.2 × 10^−^^14^ cm^3^/s—single channel permeability	Pancreas Testis	Unknown	[44,50]
AQP9	No structural data available UniProtKB—O43315 Species: *Homo sapiens* Amino acids: 295	*Xenopus* oocytes—10 ng cRNA P_f_: 12.3 × 10^−3^ cm/s (control 1.8 × 10^−3^ cm/s) P_gly_: ~10 × 10^−6^ cm/s (controls: H_2_O 0 cm/s, AQP4 ~2.5 × 10^−6^ cm/s) No single channel data available	Hepatocytes Osteoclasts Astrocytes Leukocytes	Osteoporosis	[43,51]
AQP10	PDB ref: 6F7H Resolution: 2.3 Å Species: *Homo sapiens* Method: X-ray diffraction Amino acids: 301	*Xenopus* oocytes—10 ng cRNA P_f_: ~0.05 × 10^−3^ cm/s (controls: negative control ~0.02 × 10^−3^ cm/s, AQP8 ~0.2 × 10^−3^ cm/s No single channel data available	Intestinal epithelial cells Adipocytes	Unknown	[44,52]
AQP11	No structural data available UniProtKB—Q8NBQ7 Species: *Homo sapiens* Amino acids: 271	*Spodoptera frugiperda*—Sf9 membrane vesicles containing 1 µg mouse AQP11 protein P_f_: 29.8 × 10^−3^ cm/s (controls: negative control 8.2 × 10^−3^ cm/s, 246.1 × 10^−3^ cm/s human AQP1, 197.7 × 10^−3^ cm/s His-rat AQP4) No single channel data available	Testis Thymus Kidney Liver	Polycystic kidneys	[44,53]
AQP12	No structural data available AQP12A UniProtKB—Q8IXF9 AQP12B UniProtKB—A6NM10 Species: *Homo sapiens* Amino acids: 295	Pancreatic rough endoplasmic reticulum vesicles—600 µg total RNA P_f_: 13.0 × 10^−3^ cm/s (control 15.1 × 10^−3^ cm/s) non-significant difference No single channel data available	Pancreatic acinar cells	Unknown	[54,55]

**Table 2:** Various assay methods used in the measurement of AQP function and the validation of AQP inhibitors. Their benefits and limitations are shown.

Assay Type	Assay Principle	Benefits	Limitations	References
Epithelial	Epithelial cells are placed on supports, solutes are added and transepithelial flux is measured by the height of the fluid in capillary tubes	Assists AQP identification and characterization in tissues Individual membrane permeability is calculated	Not very robust Measures paracellular as well as cellular water flow	[94,95]
Osmotic Swelling	Osmotic gradients are used to cause flux of solutes and water in cells endogenously-expressing or transfected with AQP genes; cell volume changes are measured	Well-established technique Oocytes have low intrinsic water permeability Other cells can be used Inward and outward gradients possible	Preparation techniques vary causing discrepancies in results between laboratories Not very reproducible Variability between oocytes	[94,96,98]
Microscopy	Most often fluorescent dyes are used that permeate the plasma membrane and are cleaved by esterases. As osmotic shock occurs, the fluorescence is altered, which is measured and used to calculate cell volume changes	Linear relationship produced between fluorophore and cell volume changes More accurate than counterparts Very sensitive Robust	Thickness of cell-line monolayer can produce variability in results Fluorophore may not always stay within the cell depending on cell-line	[94,99,104]
Stopped-Flow Spectroscopy	Suspensions of cells or vesicles are mixed with various osmotic solutions and flux of water causes volume changes, which result in altered light scattering. The linear relationship between optical properties and cell volume allows permeability to be calculated; alternatively fluorophores can be used.	Fast kinetics measured Can use cells, vesicles and proteoliposomes Linear relationship between optical properties and volume changes	Variability in dead time and mixing ability can cause problems with reproducibility	[94,99,101]
Computational Methods	High resolution atomic structures of AQPs are used and molecular dynamics simulations allow in silico measurement of water permeability	Provides new insights into AQP structure and function	High-quality structures not always available May disagree with experimental data	[94,103]

**Table 3:** Currently-available AQP inhibitors, their structures, the AQPs they inhibit (species are highlighted as h—human, m—mouse, and r—rat) and the conditions under which they were assayed.

Inhibitor	Conditions	AQPs Inhibited	Structure	References
Tetraethyl-ammonium	*Xenopus* oocytes 100 µM	hAQP1	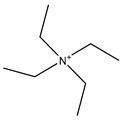	[105,106]
Phloretin	*Xenopus* oocytes 0.1 mM Proteoliposomes 0.5 mM	rAQP9 hAQP3	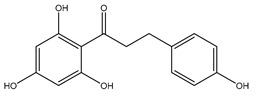	[107,108]
Mercury chloride	*Xenopus* oocytes 1 mM	hAQP1	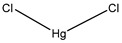	[96,109]
AuPhen	Erythrocytes 50 µM Adipocytes 15 µM	hAQP3 hAQP7	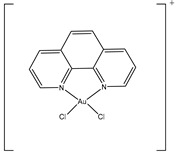	[110,111]
Silver nitrate	Erythrocytes 10 µM	hAQP3	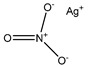	[112]
Copper sulfate	Swan 71 cells 100 µM	hAQP3	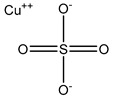	[113]
Nickel chloride	Human bronchial epithelium cells 1 mM	hAQP3	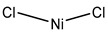	[114]
Furosemide	*Xenopus* oocytes 10 µM	hAQP1	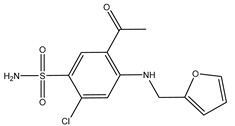	[115]
Bumetanide	*Xenopus* oocytes 100 µM	rAQP4	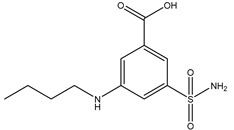	[116]
*N*-(5-Sulfamoyl-1,3,4-thiadiazol-2-yl) acetamide	*Xenopus* oocytes 20 µM	hAQP4-M23	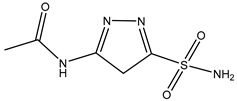	[117]
IMD-0354	Mice 0.76 mg/kg	mAQP4-M23	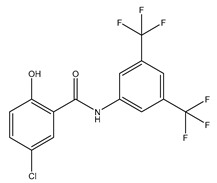	[118]
Acetazolamide	HEK293 cells 10 µM	rAQP1 rAQP4	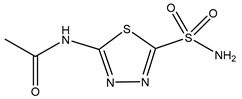	[119]
TGN-020	C57/BL6 male mice 200 mg/kg (23–28 g)	mAQP4	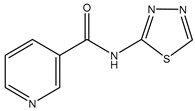	[81]
Topiramate	*Xenopus* oocytes 20 µM	rAQP4-M23	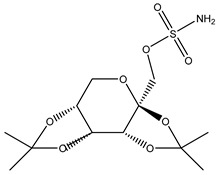	[120]
DFP00173	Human erythrocytes 25 µM	hAQP3	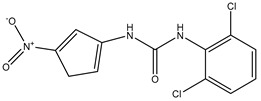	[121]
Z433927330	Human erythrocytes 25 µM	mAQP7	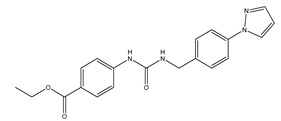	[121]

## Abbreviations

AQP	aquaporin
NPA	Asn-Pro-Ala
Å	Ångstrom
CNS	central nervous system
MIP	major intrinsic protein
RNA	ribonucleic acid
cRNA	complementary ribonucleic acid
MMP	matrix metalloproteinase
cAMP	cyclic adenosine monophosphate
PKA	protein kinase A
NDI	nephrogenic diabetes insipidus
KO	knock-out
BBB	blood–brain barrier
ICP	intracranial pressure
NMO	neuromyelitis optica
MS	multiple sclerosis
AEDs	anti-epileptic drugs
miRNA	micro ribonucleic acid
siRNA	small interfering ribonucleic acid
TEA	tetraethylammonium
µM	micromolar
HEK293	human embryonic kidney 293
MCAO	middle cerebral artery occlusion
IC50	half maximal inhibitory concentration
NiCl_2_	nickel chloride
mM	millimolar
GFP	green fluorescent protein
hAQP	human aquaporin
calcein-AM	calcein acetoxymethyl
AgNO_3_	silver nitrate
AgSul	silver sulfadiazine
IgG	immunoglobulin G
mRNA	messenger ribonucleic acid
qPCR	quantitative polymerase chain reaction
ELISA	enzyme-linked immunosorbent assay
nM	nanomolar
NF-κB	nuclear factor kappa-light-chain-enhancer of activate B cells
IKK-β	inhibitor of nuclear factor kappa-B
24h	24 hours
TBI	traumatic brain injury
mBI	mild brain injury
TNF-α	tumor necrosis factor alpha
IL1	interleukin 1
IL6	interleukin 6
UMIN	University Hospital Medical Information Network
